# Gemcitabine-Based Chemotherapy for Advanced Soft Tissue Sarcoma: Identifying the Appropriate Dose and Schedule

**DOI:** 10.7759/cureus.76149

**Published:** 2024-12-21

**Authors:** Kenji Nakano, Naomi Hayashi, Xiofei Wang, Akihiro Ohmoto, Tetsuya Urasaki, Naoki Fukuda, Yasuyoshi Sato, Makiko Ono, Junichi Tomomatsu, Mayu Yunokawa, Yuki Funauchi, Keiko Hayakawa, Taisuke Tanizawa, Keisuke Ae, Seiichi Matsumoto, Shunji Takahashi

**Affiliations:** 1 Medical Oncology, Cancer Institute Hospital of the Japanese Foundation for Cancer Research, Tokyo, JPN; 2 Genome Medicine, Cancer Institute Hospital of the Japanese Foundation for Cancer Research, Tokyo, JPN; 3 Human Oncology and Pathogenesis Program, Memorial Sloan Kettering Cancer Center, New York, USA; 4 Chemotherapy, Cancer Center, University of Tokyo Hospital, Tokyo, JPN; 5 Gynecological Oncology, Cancer Institute Hospital of the Japanese Foundation for Cancer Research, Tokyo, JPN; 6 Orthopedic Surgery, Tokyo Medical and Dental University, Tokyo, JPN; 7 Orthopedic Surgery, Cancer Institute Hospital of the Japanese Foundation for Cancer Research, Tokyo, JPN

**Keywords:** chemotherapy, docetaxel, dose modification, gemcitabine, soft tissue sarcoma

## Abstract

Background

In 2024, reimbursement for gemcitabine-docetaxel therapy (GEM-DOC; gemcitabine 900 mg/m^2^ on days 1 and 8 and docetaxel 70 mg/m^2^ on day 8 every 21 days, GEM 900-DOC 70) to treat recurrent/metastatic soft tissue sarcoma (STS) was made in Japan.

Methods

We retrospectively reviewed clinical records of advanced/metastatic STS patients who underwent off-label gemcitabine-containing chemotherapy at the Cancer Institute Hospital of the Japanese Foundation for Cancer Research between February 2007 and October 2019.

Results

Of 115 enrolled patients, 51 were treated with GEM-DOC (26 patients received the dose as previously stated) and the other 64 with gemcitabine monotherapy. Objective response rates (ORR; 9.8% versus 4.7%), progression-free survival (PFS; 3.8 months versus 2.1 months), and overall survival (OS; 21.5 months versus 11.8 months) tended to be superior in GEM-DOC; the difference of the rate of following salvage treatment (GEM-DOC, 82.4%; gemcitabine monotherapy, 48.4%) could affect the OS. As for safety, severe adverse events (grade 3 {G3} or more) were more frequent in patients with GEM-DOC (hematologic, 80.4% versus 40.6%; non-hematologic, 19.6% versus 15.6%), including those using the stated dose (hematologic, 96.1%; non-hematologic, 26.9%).

Conclusion

The stated dose of GEM-DOC could be effective for STS but shows more toxicity compared to other dose settings or gemcitabine monotherapy. An investigation of appropriate dose settings by prospective clinical trial may be needed.

## Introduction

Soft tissue sarcoma (STS) is an uncommon but not a rare cancer, accounting for approximately 1% of adult malignancy [1]. It originates from various sites of the body, and if relapsed, the prognosis is poor. Standard salvage therapy for relapsed and/or metastatic STS is systemic chemotherapy, but the efficacy is moderate, and the option of salvage chemotherapy is still limited, so new treatment options are needed.

Gemcitabine-based chemotherapy, either monotherapy or in combination with docetaxel, has been investigated for STS treatment and is regarded as a treatment option in various guidelines, but the reimbursement of gemcitabine-docetaxel (GEM-DOC) combination chemotherapy for STS had not been approved in the Japanese Health Care Insurance [2,3]. However, the reimbursement of this combination for STS has been allowed based on previous clinical data [4]. The stated dose setting is gemcitabine 900 mg/m^2^ (days 1 and 8) and docetaxel 70 mg/m^2^ (day 8) every three weeks (GEM 900-DOC 70); the dose and schedule are the same as those used in a Japanese clinical trial (JCOG1306), which evaluated the efficacy and safety of this regimen in the perioperative setting [5]; prospective data of those dose settings for recurrent/metastatic STS have been limited to a non-randomized small study [6] although there are some retrospective data of off-label use [7,8].

Thus, we collected clinical data on gemcitabine-based chemotherapy at our institute and evaluated the efficacy and safety of various regimens and, particularly, the official dose setting and combination.

## Materials and methods

We retrospectively reviewed the clinical records of advanced and/or metastatic STS patients who received gemcitabine-containing chemotherapy (gemcitabine monotherapy or combination with docetaxel) at the Cancer Institute Hospital of the Japanese Foundation for Cancer Research (Tokyo, Japan), categorizing groups by the initial dose of the regimen, and evaluated the distribution of the dose and regimen and their relationship to efficacy and safety. In particular, we collected the efficacy and safety data of the stated dose setting (GEM 900-DOC 70), which in turn was based on the dose of JCOG1306. These gemcitabine-based treatments were performed as part of clinical practice and reimbursed.

The treatment regimens were set at gemcitabine for days 1 and 8 with or without docetaxel for day 8 every three weeks, and for patients who underwent gemcitabine alone, gemcitabine for days 1, 8, and 15 every four weeks may be also allowed to be administered. Dose and regimen selection (combined with docetaxel or not) were decided based on the judgement of the attending physician. Treatment interruption, dose amendment, and granulocyte colony-stimulating factor (G-CSF) support or other supportive care were also provided for the physician’s decision. The patients’ objective responses were evaluated based on the Response Evaluation Criteria In Solid Tumours (RECIST) version 1.1. Adverse events were documented based on the Common Terminology Criteria for Adverse Events (CTCAE) version 5.0. For the evaluation of prognoses, the overall survival (OS) and the progression-free survival (PFS) were estimated by the Kaplan-Meier method. The log-rank test was used for comparing the prognoses; a comparison of response rates was performed by logistic regression analysis. For all statistical analyses, the software SPSS version 25.0 (IBM Corp., Armonk, NY) was used, and p-values of <0.05 were considered significant.

This retrospective study was designed based on the ethical guidelines for medical and biological research involving human subjects in Japan and was reviewed and approved by the Institutional Review Board of the Cancer Institute Hospital of the Japanese Foundation for Cancer Research (approval number: 2021-1041) [9].

## Results

Between February 2007 and October 2019, a total of 115 advanced/metastatic STS patients underwent gemcitabine-containing therapy; of them, 51 received combination therapy with docetaxel (of whom, 26 patients received the initial dose setting of the GEM 900-DOC 70), and the other 64 received gemcitabine monotherapy (Table 1).

**Table 1:** Characteristics of the patients enrolled in the study *Including perioperative chemotherapy n, number; %, percentage; GEM-DOC, gemcitabine-docetaxel

	GEM-DOC		GEM monotherapy	
	n	%	n	%
Age				
Median	59.5		62.3	
Range	16.4-79.8		20.2-85.4	
Gender				
Male	22	43.1	30	46.9
Female	29	56.9	34	53.1
Primary lesion				
Extremity	29	56.9	26	40.6
Non-extremity	22	43.1	38	59.4
Histology				
Leiomyosarcoma	14	27.4	12	18.8
Liposarcoma	6	11.8	10	15.6
Synovial sarcoma	2	3.9	5	7.8
Undifferentiated pleomorphic sarcoma	13	25.5	4	6.3
Other histology	16	31.4	33	51.5
Number of prior chemotherapy regimens*				
0	4	7.9	13	20.3
1	27	52.9	9	14.1
2	12	23.5	20	31.2
3	7	13.7	9	14.1
4 or more	1	2	13	20.3
Total	51		64	

**Figure 1:**
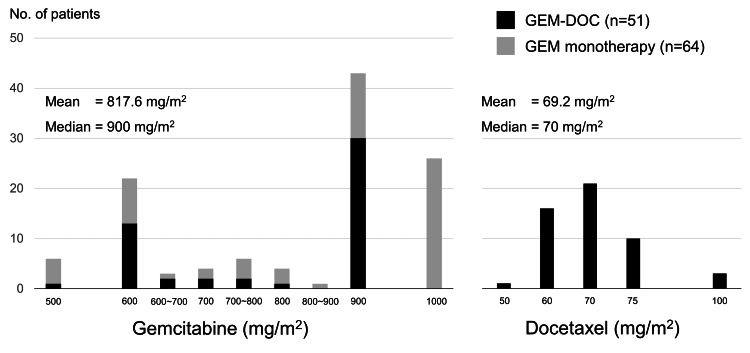
Distribution of the initial dose setting of gemcitabine and docetaxel (GEM-DOC) No: number

The initial dose distribution of each drug is shown in Figure 1; the median initial doses of gemcitabine and docetaxel were 900 mg/m^2^ and 70 mg/m^2^, respectively.

**Figure 2:**
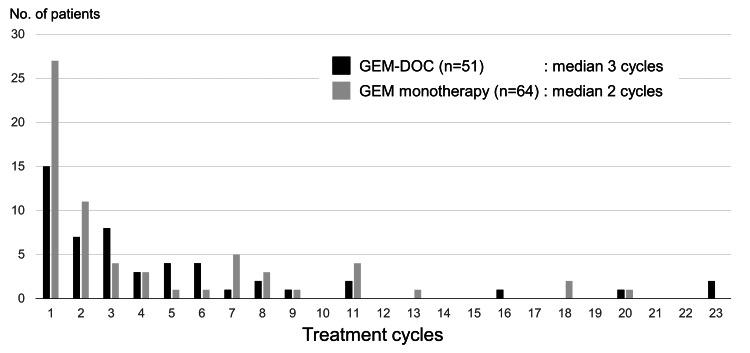
Distribution of treatment cycles of gemcitabine with or without docetaxel No, number; GEM-DOC, gemcitabine-docetaxel

The distribution of treatment cycles is shown in Figure 2; the median number of treatment cycles in patients with and without docetaxel was three and two, respectively.

Efficacy

Objective responses were observed in eight of 115 patients (6.9%), five of whom received combination therapy; thus, objective response rates (ORR) were 9.8% (5/51) in GEM-DOC and 4.7% (3/64) in GEM monotherapy. Of the 26 patients treated with GEM 900-DOC 70, objective response was observed in three patients (ORR: 11.5%).

**Figure 3:**
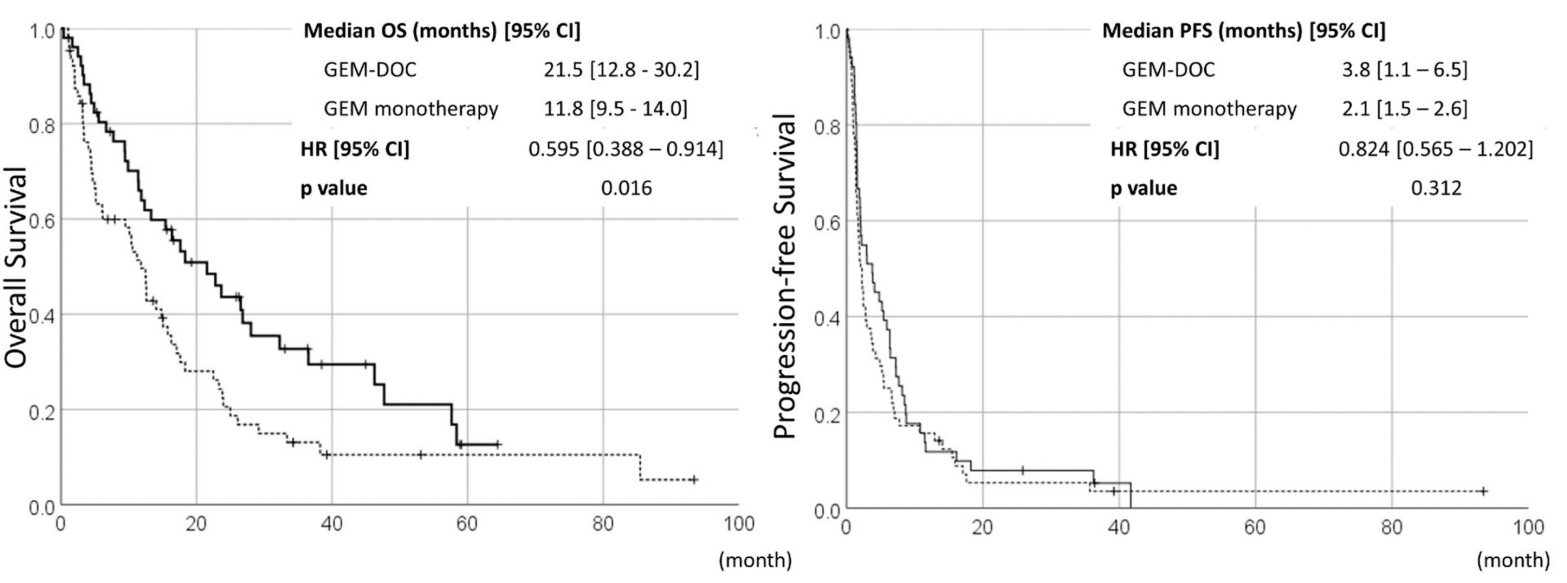
Survival curves of patients who underwent gemcitabine with or without docetaxel OS, overall survival; PFS, progression-free survival; GEM-DOC, gemcitabine-docetaxel

In terms of prognoses, the overall survival (OS) tended to be longer in patients with docetaxel (p=0.016), and there was even less of a difference in progression-free survival (PFS) between patients with and without docetaxel (Figure 3).

Details of posttreatment after GEM-based chemotherapy are summarized in Table 2.

**Table 2:** Details of anticancer treatments following GEM-based chemotherapy *Etoposide in nine patients, carboplatin in seven patients, ifosfamide in five patients, and doxorubicin, dacarbazine, vincristine, actinomycin-D, cyclophosphamide, and clinical trial enrollment in one patient each **Carboplatin in five patients, etoposide in four patients, doxorubicin and clinical trial enrollment two patients each, and ifosfamide in one patient GEM-DOC: gemcitabine-docetaxel

	GEM-DOC (n=51)		GEM monotherapy (n=64)	
	n	%	n	%
None	9	17.6	33	51.6
Any	42	82.4	31	48.4
Surgery	9	17.6	4	6.3
Radiation	19	37.3	15	23.4
Chemotherapy	34	66.7	22	34.4
Pazopanib	21	41.2	16	25
Eribulin	14	27.5	5	7.8
Trabectedin	6	11.8	1	1.6
Others	13*	25.5	8**	12.5

**Figure 4:**
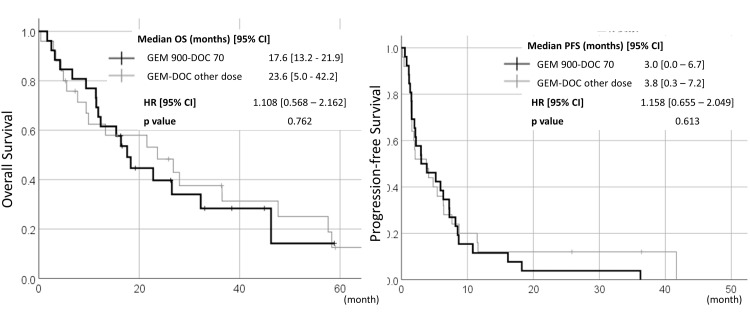
Survival curves of patients who underwent gemcitabine with docetaxel at the dose of JCOG1306 (gemcitabine 900 mg/m2 on days 1 and 8 and docetaxel 70 mg/m2 on day 8 every three weeks, GEM 900-DOC 70) or at another setting OS, overall survival; PFS, progression-free survival

Any antineoplastic therapy was received 82.4% (42/51) in the GEM-DOC group and 48.4% (31/64) in the GEM monotherapy group, with rescue surgery, radiation therapy, and chemotherapy being performed more frequently in the GEM-DOC group. Within patients undergoing docetaxel-combined chemotherapy, there were no differences in prognosis (OS or PFS) between GEM 900-DOC 70 and other dose settings (Figure 4).

Safety

The frequency of major hematologic and non-hematologic adverse events is summarized in Table 3 and Table 4. Hematologic adverse events were more frequently observed in patients taking gemcitabine combined with docetaxel (Table 3); among the combination-therapy patients, those using GEM 900-DOC 70 had more frequent adverse events than those with other dose settings (Table 4).

**Table 3:** Adverse events (AEs) in GEM-DOC therapy and GEM monotherapy GEM-DOC: gemcitabine-docetaxel

	GEM-DOC (n=51)				GEM monotherapy (n=64)			
	All grade		Grade 3 or more		All grade		Grade 3 or more	
	n	%	n	%	n	%	n	%
Hematologic			41	80.4			26	40.6
Leukopenia	47	92.2	38	74.5	34	53.1	12	18.8
Neutropenia	46	90.2	35	68.6	32	50.0	18	28.1
Anemia	47	92.2	10	19.6	44	68.8	9	14.1
Thrombocytopenia	41	80.4	17	33.3	29	45.3	7	10.9
Febrile neutropenia	12	23.5	9	17.6	2	3.1	2	3.1
Non-hematologic			10	19.6			10	15.6
Nausea/vomiting	21	41.2	1	2	18	28.1	0	0
Diarrhea	7	13.7	0	0	3	4.7	0	0
Constipation	17	33.3	1	2	23	35.9	1	1.6
Fever	8	15.7	0	0	13	20.3	0	0
Edema	9	17.6	0	0	6	9.4	0	0
Fatigue	22	43.1	0	0	12	18.8	0	0
Skin rash	13	25.5	1	2	8	12.5	1	15.6
Neuropathy	11	21.6	0	0	2	31.3	0	0
Total bilirubin increased	9	17.6	1	2	7	10.9	0	0
Transaminase increased	32	62.7	3	5.9	41	64.1	5	7.8
Serum creatinine increased	10	19.6	0	0	16	25.0	0	0
Other severe AEs	6	11.8	6	11.8	5	78.1	5	7.8

**Table 4:** Adverse events (AEs) in GEM 900-DOC 70 and other GEM-DOC doses GEM 900-DOC 70, gemcitabine 900 mg/m^2^ on days 1 and 8 and docetaxel 70 mg/m^2^ on day 8 every three weeks; GEM-DOC, gemcitabine-docetaxel

	GEM 900-DOC 70 (n=26)				Other GEM-DOC doses (n=25)			
	All grade		Grade 3 or more		All grade		Grade 3 or more	
	n	%	n	%	n	%	n	%
Hematologic			25	96.1			16	25.0
Leukopenia	26	100	23	88.5	21	84.0	15	60.0
Neutropenia	26	100	20	76.9	20	80.0	15	60.0
Anemia	25	96.1	7	26.9	22	88.0	3	12.0
Thrombocytopenia	24	92.3	12	46.2	17	68.0	5	20.0
Febrile neutropenia	9	34.6	7	26.9	3	12.0	2	8.0
Non-hematologic			7	26.9			3	12.0
Nausea/vomiting	10	38.5	0	0	11	44.0	1	4.0
Diarrhea	5	19.2	0	0	2	8.0	0	0
Constipation	9	34.6	1	3.8	8	32.0	0	0
Fever	7	26.9	0	0	1	4.0	0	0
Edema	6	23.1	0	0	3	12.0	0	0
Fatigue	14	53.8	0	0	8	32.0	0	0
Skin rash	9	34.6	1	3.8	4	16.0	0	0
Neuropathy	6	23.1	0	0	5	20.0	0	0
Total bilirubin increased	3	11.5	0	0	6	24.0	1	4.0
Transaminase increased	17	65.4	1	3.8	15	60.0	2	8.0
Serum creatinine increased	4	15.4	0	0	6	24.0	0	0
Other severe AEs	4	15.4	4	15.4	2	8.0	2	8.0

## Discussion

Gemcitabine-based chemotherapy for STS was first introduced in the publication of a phase 2 trial in the early 2000s by Hensley et al., in which the efficacy and safety of the combination of gemcitabine and docetaxel were evaluated [10]. A total of 34 patients were enrolled (29 of whom had uterine leiomyosarcoma), and the ORR was observed as 53% (95% CI: 35-70). Since then, several clinical trials of gemcitabine-based chemotherapy for STS have been undertaken and published [11,12]; these formed the basis of the guideline dose and schedule [2,3]. Since uterine leiomyosarcoma was a criterion of enrollment in Hensley et al.’s trial [13], gemcitabine-based chemotherapy was particularly of interest in gynecological settings [6], but following clinical trials have shown lower response rates than those reported by Hensley et al., and the results of recent domestic and international clinical trials suggest that response rates to gemcitabine-based chemotherapy are generally expected to be around 10%-20% [13].

Despite energetic investigations, solid evidence regarding gemcitabine-based chemotherapies for STS has not been established: in both the perioperative and recurrent/metastatic settings, gemcitabine-based chemotherapy has failed to show survival benefits in phase 3 trials [5,14]. Another problem has been that the dose, schedule, and/or regimen (gemcitabine monotherapy or combination with other antitumor drugs such as docetaxel) have been different in each clinical trial: no global consensus on the appropriate dose setting has yet been established. Thus, gemcitabine-based chemotherapy has not been officially approved by the FDA, European Medicines Agency (EMA), and other regulatory authorities although many guidelines identify it as a treatment option [2,3].

In Japan, the 1980 notification from the Director of the Health Insurance Bureau at the Ministry of Health, Labor and Welfare (MHLW) was applied to the off-label use of drugs to treat rare diseases including some types of cancer, and the validity of the reimbursement of gemcitabine-based regimen for STS had been discussed and certified on a case-by-case basis [15]. Since the 2010s, as the movement toward the unification of reimbursement criteria progressed, the discussion of the indication and validity for gemcitabine-based STS treatment regimens was renewed [16]. Finally, based on the results of the clinical use to date and the results of domestic and international clinical trials, the Health Insurance Claims Review and Reimbursement Services was notified that reimbursement would be allowed in 2024 [4]. The stated dose and schedule setting was certified as GEM 900-DOC 70, based on JCOG1306, the largest prospective clinical trial, and this can be expected to be the standard in Japan. However, the JCOG1306 trial evaluated the efficacy of GEM 900-DOC 70 only as a perioperative adjuvant chemotherapy, and there is still a lack of data to verify GEM 900-DOC 70 in terms of efficacy, let alone safety. So, in this retrospective analysis, we examined the validity of a gemcitabine-based regimen as a salvage therapy for recurrent or metastatic STS, including the benefits and risks of the docetaxel combination and the stated dose.

The efficacy of GEM-based regimens in our retrospective analysis is similar to those of recent prospective/retrospective data in Japan [5-8]. Combination with docetaxel showed improved ORR but not PFS: the same result as in previous randomized phase 2 trials [11,12]. The OS was longer in patients with docetaxel-combined therapy. However, reviewing the background of enrolled patients, the GEM-DOC cohort included 60.1% (31/51) of patients with zero to one previous chemotherapy regimens, while those in the GEM monotherapy cohort were 34.4% (22/64), so the OS difference between GEM-DOC and GEM monotherapy could derive from the differences in the number of previous/posttreatment of the two groups. In terms of dose setting in GEM-DOC cohort patients, there were no apparent differences in efficacy between GEM 900-DOC 70 and other dose settings.

As for safety, a higher frequency of adverse events was reported in the GEM-DOC combination cohort; most of these cases experienced hematologic toxicity. Hematologic toxicity was particularly more frequent in the GEM 900-DOC 70 patients, of whom all had grade 3 (G3) or higher myelosuppression. This corresponds with the increased frequency of myelosuppression detected in patients treated with GEM 900-DOC 70 in JCOG1306, which strictly followed the laboratory data during treatment. The frequency of G3 or higher hematologic toxicity in JCOG1306 was 82.2% [5]; the difference in frequency between JCOG1306 and the present analysis could be explained by the difference in patient backgrounds of adjuvant therapy and recurrent/metastatic cases.

Since there was no difference in the efficacy compared to other dose settings and no cases of treatment-related death due to adverse events, GEM 900-DOC 70 can be considered to be a feasible dose with appropriate supportive care and adverse event management. The higher response rate in the GEM-DOC combination compared to GEM monotherapy makes the GEM-DOC a better choice for patients tolerable to treatment, but the lower frequency of adverse events in the GEM group suggests that GEM monotherapy may be considered for heavily treated patients to avoid potentially lethal adverse events.

By the current evidence, GEM-DOC has not shown superiority to doxorubicin for first-line therapy in the phase 3 trial [14] and is considered a second- or later-line therapy option, but its clinical benefit compared to other drugs, especially those newly approved since the 2010s (such as pazopanib, trabectedin, and eribulin), is not clear [17-19]. A randomized controlled trial comparing the efficacy of rescue therapies in second-line treatment is underway in Japan, and it is hoped that a prospective randomized controlled trial will clarify the clinical position of GEM 900-GOC 70 in the future [20,21].

There are some limitations in our study: retrospective design, non-randomized allocation to the regimen (combined with docetaxel or not), and the decision of dose setting and modification mainly made by physicians’ decision, not by unified protocol.

## Conclusions

We report a retrospective study of the efficacy of GEM-based chemotherapy in advanced/metastatic STS patients, and the results of efficacy and safety were similar to those reported in previous studies in Japan and international clinical trials.

In the stated dosage regimen of GEM 900-DOC 70, used in a Japanese clinical trial and the approved dose setting for reimbursement, a relatively high response rate was expected due to the combination with docetaxel, but the frequency of adverse events was high, especially hematologic toxicity. It is considered that further study of the clinical positioning and appropriate dosage setting is needed.
